# Morbidity assessment of *Opisthorchis viverrini* infection in rural Laos: I. Parasitological, clinical, ultrasonographical and biochemical findings

**DOI:** 10.1186/s41182-016-0012-y

**Published:** 2016-05-16

**Authors:** Hermann Feldmeier, Miklos Hazay, Megumi Sato, Pongvongsa Tiengkham, Futoshi Nishimoto, Hongwei Jiang, Vatsana Sopraseuth, Kazuhiko Moji

**Affiliations:** Institute of Microbiology and Hygiene, Charité University Medicine, Berlin, Germany; Specialist Practice for Internal Medicine, Hamburg, Germany; Graduate School of Health Sciences, Niigata University, Niigata, Japan; Malaria, Parasitology and Entomology Section, Savannakhet Province, Lao PDR; Graduate School of Tropical Medicine and Global Health, Nagasaki University, Nagasaki, Japan; Research Institute for Humanity and Nature, Kyoto, Japan; Savannakhet Provincial Hospital, Savannakhet Province, Lao PDR

## Abstract

**Background:**

Infections with the food-borne trematode *Opisthorchis viverinni* are common in Southeast Asia. In Lao PDR alone, two million people are supposed to be infected. Opisthorchiasis may cause severe liver disease, eventually leading to cholangiocarcinoma. The objective of this study is to assess the eating habits, complaints, symptoms, signs and ultrasonographical findings in three different areas of Savannakhet Province.

**Methods:**

Study participants were recruited in Lahanam village in the flood-prone lowland of, Sonkhone district, Savannakhet Province (group A); in Non Somboon village, a community located on a hilly plateau in the same district (group B); and in staff of Savannakhet Province Hospital, Savannakhet town (group C). Eating habits, complaints and symptoms were recorded by standardized structured questionnaires. Participants were thoroughly examined clinically, and ultrasonography was performed. *O. viverrini* eggs were looked for in stool and in duodenal fluid. An array of biochemical and haematological parameters potentially related to liver disease was determined. Group A consisted of 45, group B of 31 and group C of 18 individuals.

**Results:**

Eating habits were similar in the three groups, except that participants from group C tended to consume less high-risk types of fish dishes and more frequently ate beef and pork. Average intensity of infection (eggs per gram of stool) was low, but significantly higher in group A than in group B and C (*p* < 0.001). Medical history and complaints were similar in the three groups. Ultrasonography did not reveal any bile duct pathology. The only pathological finding was a slight elevation of ASAT and gamma-GT in a few participants in groups A and B.

**Conclusions:**

The study shows that eating habits favouring the infection with *O. viverrini* are common in south Laos. Although the average intensity of infection was low, there was a significant difference between the groups, paralleling slightly different eating habits. Clinically, this corresponded to a paucity of liver disease-associated complaints and signs. The low intensity of infection probably explains why no alterations of bile ducts were detectable by ultrasonography.

## Background

The oriental liver flukes (*Opisthorchis viverrini*, *Opisthorchis felineus*, *Chlonorchis sinensis*) are important causes of human disease. In 2005, the total number of individuals infected with *Opisthorchis* spp*.* alone was estimated to be 8.4 million [1]. In Southeast Asia, opisthorchiasis alone was responsible for 63,000 of years of life lost and 74,000 disability-adjusted life years (DALYS) [1]. In the 1980s, the prevalence of *O. viverrini* infection in Northeast Thailand was as high as 90 % in the general population [2]. In Cambodia, in a population-based study, the prevalence was 32 % in 2000 [3]. In Lao PDR, two million people are supposed to be infected, prevalence being particularly high in the central and southern lowlands [4]. A nationwide parasitological survey of 29,846 primary school children showed an average prevalence of 10.9 % and a maximum of 32.2 [4]. The prevalence of *O. viverrini* in Sanvannakhet Province is estimated to be 36 % (Pongvongsa Tiengkham, unpublished observation 2008). There is reason to assume that liver fluke disease occurs in all parts of the country, even in the surroundings of Vientiane [5]. Presumably, prevalence of human infection essentially depend on the population’s eating customs [6].

Infection is acquired by the ingestion of raw or inadequately cooked infected freshwater fish or by semi-fermented or pickled fish flesh. Adult *O. viverrini* flukes may live up to 20 years in the bile ducts of their final hosts. One female fluke lays 2000–4000 eggs each day. Eggs pass down the bile duct into the intestine but can also be found in the gallbladder. In opisthorchiasis, the flukes reside mainly in the small- and medium-sized bile ducts [7].

In the endemic area, opisthorchiasis comprises a broad array of morbidity, clinical pathology usually being absent or minimal in light infection with a tendency to become more and more severe when worms accumulate in a host over time [6]. However, even in intensely infected patients, haematological and biochemical measurements may remain unremarkable [2, 8]. Similarly, ultrasonography may or may not show morphological abnormalities [9]. The objective of this study was to assess clinical, biochemical and immunological markers of *O. viverrini*-associated morbidity in individuals sharing the same ethnicity and cultural background, but living in different micro-environments in Savannakhet Province. The immunological investigations are reported in a companion manuscript.

## Methods

### Study area and study population

The study was carried out in two villages in Sonkhone district, Savannakhet Province in southern Lao PDR as well as in Savannakhet town. Lahanam is located at the Banghiang river, a tributary of Mekong river, about 70 km upstream from the confluence into the Mekong river (16° 16′ North, 105°, 10′ East). Lahanam is a traditional village of Pu-Thai ethnic group and comprises about 2200 inhabitants. People mainly live from paddy farming, animal husbandry, weaving and income earned by migrant workers in Thailand. Eating habits are deeply rooted in local culture and include a variety of meals and dishes putatively containing infectious metacercariae such as *Koi-pa dip* (chopped raw fish), *Laap-pa dip* (raw fish mixed with vegetables) and *Laap-sa pa* (fish heated in water at 50–80 °C for approximately 30 s) [10]. Participants of Lahanam village constituted group A. Non Somboon is a community located on a hilly plateau at the main road linking Vientiane to Pakse. Since the village is located at the main road, it is more affluent than Lahanam. People mainly live from paddy farming, animal husbandry, weaving and income earned by migrant workers in Thailand. Participants from Non Somboon village constituted group B. The third group consisted of medical doctors, nurses and technicians of Savannakhet Province Hospital, Savannakhet town. In the study area, praziquantel mass treatment was never performed. Whether single individuals have been treated with praziquantel is not known. In view of the limited availability of praziquantel, this is rather unlikely. All participants denied having been treated with praziquantel.

### Study design

Participants were recruited on a voluntary basis. In group A, the first 50 individuals aged >20 years excreting *O. viverrini*-eggs were identified during a community-based coprological survey and were invited to participate at the study provided they were willing to swallow a duodenal probe and to undergo an ultrasound examination. The second inclusion criterion was residence in Lahanam village for at least 15 years with a maximal interruption of 2 years. Age >20 years was set as an inclusion criterion, since intensity of infection starts to peak at this age [1].

In Non Somboon village, the study was announced through traditional community leaders and the local government. The first 35 individuals were included in the study on a first-come-first-serve basis provided they were willing to provide three stool samples and to undergo a clinical examination including ultrasonography. In Savannakhet Province Hospital, the study was announced through the director of the hospital, and technicians and nurses were invited to participate, irrespective to which department they belonged, provided they were willing to provide three stool samples and to undergo a clinical examination including ultrasonography.

All individuals identified as being infected with *O. viverrini* were treated with praziquantel immediately after the diagnosis was established. Praziquantel was administered in a single dose of 40 mg/kg. If intestinal helminths were detected, those were treated according to the recommendations of the Ministry of Health.

### Eating habits

A structured interview in Lao language was performed to collect data on eating habits. Questions concerned the type of food participants usually consumed, the way fish was prepared (boiled, roasted, semi-fermented, fermented, raw mixed with vegetables, raw with chilli sauce for dipping, etc.) and where the consumed fish originated from.

### Clinical examination and ultrasonography

Each participant was carefully examined. Special attention was given to jaundice, palmar erythema, spider naevi and other indicators of collateral vascularization. Medical history and complaints were assessed using a standardized questionnaire. Questions were translated from English into Lao by a bilingual speaker. A complaint score was constructed in the following way: general health feeling; nausea; vomiting; diarrhoea; abdominal discomfort; feeling of fullness; indigestion; bad-smelling stool; pain upper right quadrant during the last 3-months: 0, 1 or 2 points, respectively (well/never, not good/sometimes, feeling weak/regularly, respectively); weight loss and loss of appetite during last 3 months: 0 or 1 point (yes/no). The score has a minimum of 0 and a maximum of 20 points.

Ultrasonography was performed with a portable scanner (VScan, General Electric Company, Fairfield, CT, USA). Special attention was given to non-shadowing echogenic foci or cast signs (considered to be characteristic for *O. viverrini*-related bile duct abnormalities) and floating or dependent discrete echogenic foci or swirling shadows after applying pressure to the abdomen (signs indicating the presence of worms in the gallbladder).

### Laboratory examinations

Vacutainers (Becton, Dickinson & Company, Heidelberg, Germany) were used to draw 10 ml of non-anticoagulated and 5 ml of EDTA-anticoagulated venous blood. Serum was aliquoted into 2-ml samples immediately after centrifugation and stored at −60 °C. Haemoglobin, haemotocrite and leucocyte counts were determined in Savannakhet Province Hospital. A differential white blood cell count was performed. Alkaline phosphatase (ALT), glutamate pyruvate transaminase (ALAT), aspartate transaminase (AST), gamma-glutamyltransferase (gamma-GT) lipase and bilirubin were determined using commercially available kits. To differentiate between normal and pathological values, the cut-offs defined by the Ministry of Health Lao PDR were used.

### Duodenal fluid

Duodenal fluid for the detection of *O. viverrini* eggs was collected and processed as previously described [11]. In brief, a flexible tube was inserted through the nose. The tip of the tube was coated with an anaesthetic gel to facilitate swallowing. When the tube had entered the duodenum—as indicated by a pH-change from 2 to 7—50 ml of ice-cold water was instilled. When ultrasonography showed that the gallbladder had contracted, the instilled water was re-aspirated. If the gallbladder did not contract after the first instillation of ice-cold water, the procedure was repeated a second time. When bile was aspirated, the tube was connected to a urine collection bag. The bag was fixed at the lower part of the examination couch so that bile fluid could drain into the bag. Bile fluid was collected for a maximum of 1 h.

The collected fluid was filtered through a polycarbonate membrane of 25-mm diameter and 8-μm pore size (Millipore, Massachusetts, USA), as previously described [11]. If the fluid was heavily contaminated with mucine (leading to rapid clogging of the filter membrane), a few drops of a solution of 0.1 g dithiothreitol in distilled water were added (Axis Shield Diagnostics, Dundee, UK). Eggs present on the membrane were counted at ×400 magnification.

The method allows the unambiguous detection of *O. viverrini* eggs, since eggs detected on the membrane can be clearly identified [11]. In stool, eggs are confounded with similarly looking eggs from intestinal trematodes (see below). Since this method is invasive and stressful for the patient, it was only performed in participants of group A, i.e. in those individuals in which the diagnosis *O. viverrini* infection was already confirmed by stool examination.

### Stool

Of each patient, three stool samples were collected on three subsequent days. Kato-katz thick smears were prepared using a commercially available kit (Department of Helminthology, Mahidol University, Bangkok, Thailand). The number of eggs per gram of stool was calculated for each smear, and the arithmetic mean of the three smears was calculated.

Since even an experienced microscopist cannot differentiate between O. *viverrini* eggs and eggs of intestinal trematodes of *Haplorchis* spp. and lecithodendriid flukes, such as *Prosthodendrium molenkampi* and *Phaneropsolus bonnei*, it cannot be excluded that a Kato-Katz result might contain a certain number of eggs from intestinal trematode species.

### Statistics

Ordinal variables were compared with the Mann-Whitney text. The Spearman rank correlation coefficient text was used to assess the correlation between ordinal variables. Relative frequencies were compared with the chi-square test. All analyses were performed using Sigmaplot Version 10.0 (Systat Software Inc, USA). Estimating a difference of 25 % between the average intensity of infection between group A and groups B and C, forty-five individuals had to be included in each of the groups (probability = 0.05, power of the test = 0.80). However, neither in group B nor in group C this sample size was achieved.

### Ethical considerations

The study was performed according to good clinical practice and in accordance with the Declaration of Helsinki as amended in 2008. The study was approved by the Ethics Committee of the Research Institute for Humanity and Nature, Kyoto, Japan, and the National Ethics Committee for Health Research, Ministry of Health, Lao PDR. Before the clinical examination, the objectives of the study and the procedures foreseen were explained in Lao language and informed written consent was obtained. The participants were informed that they could withdraw from the study at any time, particularly if they felt uncomfortable with the swallowed probe. After the probe was removed, the patients remained in a room under supervision of a physician for 2 h.

After the examination, each participant received a food basket containing rice and snacks and was offered a hot meal. Within 3 days, all participants were informed about key results of the laboratory examinations. All data were entered into an Excel data base and anonymized for later analysis.

## Results

### Demography

Demographic data are shown in Table 1. Patients of group A and B were of similar age (median 40 and 43 years, respectively), while those of group C were considerably younger (median age 33 years). The male-female ratio was 1.6 in group A and 0.8 in groups B and C. Whereas the height was identical in all groups, participants of group A weighted less than those of groups B and C (53 versus 57 and 58 kg, respectively). However, the difference was not significant.

**Table 1:** Demographic characteristics of the participants

	Group A (*n* = 45)	Group B (*n* = 31)	Group C (*n* = 18)
Sex			
Male	28	14	8
Female	17	17	10
Age			
Median	40	43	33
Range	20–58	26–56	22–43
Height (cm)			
Median	155	155	155
Range	143–170	143–175	148–172
Weight (kg)			
Median	53	57	57
Range	39–78	40–79	46–77

### Eating habits

Eating habits in the three groups are summarized in Table 2. Ninety-five percent of the participants of group A regularly ate fish (once a week or almost daily). Beef and pork were very rarely consumed. Mostly, fish was consumed in raw or semi-raw manner. Raw fish mixed with vegetables and fermented fish were favourite dishes of the patients. In group B, eating habits were similar, except that only 29.9 % of the participants said that they ate fish almost every day (group A = 67.5 %; *p* = 0.02). Participants of group B also cited raw fish mixed with vegetables as the preferred dish (61 %; group A 67.5 %; not significant). In group C, there was a tendency in consuming fish preferably boiled, deep-fried or dried. Participants in group C ate more often beef and pork than those of the other groups. However, 22 % consumed raw fish mixed with vegetables at least from time to time.

**Table 2:** Eating habits in the three groups

Eating habit	Group A (*n* = 40)	Group B (*n* = 31)	Group C (*n* = 18)
Type of food consumed	A/B/C/D (%)	A/B/C/D (%)	A/B/C/D (%)
Fish from pond or paddy field	0.0/5.0/27.5/67.5	0.0/35.5/35.5/29.9	0.0/28.0/11.0/61.0
Snail from pond or paddy field	7.5/72.5/17.5/2.5	10.0/80.0/10.0/0.0	44.0/56.0/0.0/0.0
Shrimp from pond or paddy field	32.5/67.5/0.0/0.0	19.0/68.0/10.0/3.0	17.0/78.0/6.0/0.0
Frog from pond or paddy field	5.0/55.5/25.0/15.0	3.0/58.0/35.5/3.5	28.0/67.0/0.0/0.0
Beef	0.0/80.0/12.5/7.5	0.0/59.0/45.0/0.0	0.0/17.0/39.0/44.0
Pork	2.5/75.0/15.0/7.5	22.5/58.0/19.5/0.0	0.0/6.0/39.0/56.0
Fish from pond/paddy field is eaten^a^	%	%	%
Boiled	72.5	94.0	67.0
Roasted	77.5	26.0	67.0
Deep-fried	12.5	16.0	22.0
Raw with chilli sauce for dipping	32.5	6.5	33.0
Raw together with vegetable	67.5	61.0	22.0
Cooked together with vegetable	17.5	13.0	56.0
Semi-fermented^b^	34.5	3.2	5.6
Fermented^c^	97.5	3.2	28.0
Dried	32.5	94.0	0

*A* never, *B* rarely, *C* once a week, *D* almost every day

^a^Multiple mentions possible

^b^Raw fish is fermented a couple of days

^c^Raw fish is fermented a couple of weeks or up to one year

### Medical history and complaints

Medical history and current complaints were similar in the three groups (Table 3). Only the rather vague complaint “feeling bad” was more frequently noted in group A than in groups B and C (24.4, 9.4 and 11.1 %, respectively; *p* = 0.005). Weight loss during the last 3 months was more frequently observed in group A than in groups B and C (40, 32.2 and 5.6 %, respectively; not significant).

**Table 3:** Medical history and complaints in the three groups

Complaints during the last 3 months	Group A (*n* = 45) %	Group B (*n* = 31) %	Group C (*n* = 18) %
General health feeling	A/B/C^a^	A/B/C^a^	A/B/C^a^
	31.2/44.4/24.4	48.7/41.9/9.4	55.6/33.3/11.1
Nausea	D/E/F^b^	D/E/F^b^	D/E/F^b^
	55.6/42.2/2.2	71.0/29.0/0.0	55.6/44.4/0.0
Vomiting	91.2/8.8/0	83.9/16.1/0.0	88.9/11.1/0.0
Diarrhoea	73.3/26.7/0	77.4/16.1/6.5	88.9/0.0/11.1
Abdominal discomfort	46.7/46.7/6.6	25.8/35.5/38.7	38.9/61.1/0.0
Feeling of fullness	44.5/53.3/2.2	87.0/6.5/6.5	38.9/38.9/22.2
Indigestion	64.5/33.3/2.2	48.4/41.9/9.7	77.8/22.2/0.0
Bad smelling stools	60.0/37.8/2.2	57.6/29.0/13.4	72.2/27.8/0.0
Pain upper right quadrant	77.8/20.0/2.2	38.7/0.0/61.3	66.6/5.6/27.8
Loss of appetite	No/Yes	No/Yes	No/Yes
	86.7/13.3	35.5/64.5	94.4/5.6
Weight loss	60.0/40.0	67.8/32.2	94.4/5.6
Complaint score^c^ median (range)	5 (0-12)	7 (0-11)	4 (0-10)

^a^ *A* good, *B* not good, *C* bad

^b^ *D* never, *E* sometimes, *F* regularly

^c^See “Methods” section

The complaint score varied over a wide range. However, the median did not differ between the groups. In none of the groups, the complaint score correlated with the intensity of *O. viverrini* infection.

### Clinical findings and ultrasonography

One participant of group A showed icterus, a palpable liver margin, pain upon pressure in the upper right quadrant, ascites and oedema. This patient excreted 600 *O. viverrini* ova per gram. Another participant of this group had pain upon pressure in the right upper quadrant. The examination of all other participants of this group was uneventful. In none of the participants of groups B and C, pathological signs were detected.

The ultrasound examination was unrevealing in all groups. The liver size and the size of the gallbladder were also normal. The bladder wall did not show signs of a thickening or polyps. In a single participant of group A, gallbladder stones were detected. In another participant of group A, there was presence of sludge in the gallbladder. Floating or dependent discrete echogenic foci or swirling shadows after applying pressure (signs indicating the presence of worms in the gallbladder) were not observed. In all cases, the intrahepatic and the extrahepatic biliary ducts were normal. Intrahepatic stones were not detected. In intrahepatic and extrahepatic biliary ducts, non-shadowing echogenic foci or casts were not detected. The pancreas was normal in all cases.

### Parasitological data

Parasitological findings in stool and duodenal fluid are summarized in Table 4. The overall intensity of infection was low, but the pattern differed between the groups. In group A, the median egg excretion of *O. viverrini* in stool was 900 eggs per gram; (range 60–13,710 eggs per gram (epg)). Six participants (13.3 %) had a high intensity of infection (>6000 epg). *O. viverrini* eggs were detected in 19/32 (59.4 %) of duodenal fluid samples, the intensity varying widely (range 1–7140; median 24).

**Table 4:** Parasitological findings in the three groups

	Group A (*n* = 45)				Group B (*n* = 31)		Group C (*n* = 18)	
	Stool		Duodenal fluid		Stool			
	Kato-Katz		Filtration		Kato-Katz		Kato-Katz	
Helminth species	Positive/examined (%)	Intensity (epg)	Positive/examined (%)	Intensity^b^	Positive/examined (%)	Intensity (epg)	Positive/examined (%)	Intensity (epg)
		Median (range)		Median (range)		Median (range)		Median (range)
*Opisthorchis viverrini*	45/45 (100)	900 (60–13,710)	19/32 (59.4)	24 (1–7140)	15/29 (52.7)	40 (24–56)	9/18 (50.0)	24 (24–40)
Hookworm^a^	18/45 (40.0)	180 (30–2940)	4/32 (12.5)	13 (5–27)	6/29 (20.7)	24 (24–72)	0/18 (0)	n.a.
*T. trichiura*	4/45 (8.9)	n.d.	0/32 (0)	n.a.	0/29 (0)	n.d.	0/18 (0)	n.a.
*Taenia* spp*.* ^a^	7/45 (15.6)	n.d.	0/32 (0)	n.a.	0/29/0)	n.a.	0/18 (0)	n.a.
*Trichostrongylus* spp*.* ^b^	0/45 (0)	n.a.	0/32 (0)	n.a.	3/29 (10.3)	n.d.	2/18 (11.1)	n.d.

*n.a.* not applicable, *n.d.* not determined

^a^Species not identified

^b^Number of eggs in the total volume of duodenal liquid filtrated

In group B, 15/29 (52.7 %) participants excreted *O. viverrini* in stool and in group C 9/18 (50.0 %). However, egg excretion in those positive was low to very low (median 40 and 24, respectively; note: 24 epg correspond to the lowest number of eggs detectable by a Kato-Katz smear). Intensity of infection was significantly higher in group A compared to groups B and C (both *p* < 0.001). The difference of intensity of infection between groups B and C was at the limit of statistical significance (*p* = 0.055).

Hookworm eggs were detected in 40.0 % in participants of group A. The number of hookworm eggs in stool was on average five times lower than of *O. viverrini* eggs. Other food-borne trematodes and nematodes (*Taenia* spp., *Trichostrongylus* spp., *Trichuris trichiura)* were rare. Hookworm eggs were detected in 6/29 (20.7 %) of the participants of group B, but none of group C.

### Haematology

Haematological data are summarized in Table 5. Slight anaemia was present in 26.7 % of the participants in group A and in 38.7 % and in 27.7 % of group B and C, respectively (difference not significant). In groups A and B, anaemia was more frequent in males than in females, although the difference was not significant. In contrast, in group C, the opposite was true.

**Table 5:** Haematological findings in the three groups

	Group A (*n* = 45)		Group B (*n* = 31)		Group C (*n* = 18)	
Variable	Median (range)	Outside normal range^a^ (%)	Median (range)	Outside normal range^a^ (%)	Median (range)	Outside normal range^a^ (%)
Haematocrite (%)						
Male	42.0 (33.8–47.1)	12/28 (42.8)	40.4 (36.7–46.3)	8/14 (57.1)	44.0 (32.4–53.6)	2/7 (28.6)
Female	37.8 (32.8–43.2)	6/17 (35.2)	36.8 (31.1–42.3)	9/17 (53.0)	34.6 (28.3–41.3)	8/11 (72.7)
Haemoglobin (g/dl)						
Male	14.4 (10.9–16.4)	10/28 (35.7)	13.5 (11.7–16.8)	7/14 (50.0)	14.9 (10.2–16.6)	1/7 (14.3)
Female	13.0 (11.2–14.3)	2/17 (11.7)	12.4 (10.5–14.3)	5/17 (29.4)	12.0 (9.1–13.7)	4/11 (36.4)

^a^Normal values and definitions: Haematrocrite: males 40.9–49.8 %, females 36.8–43.7 %; Haemoglobin: males 13.5–16.5 g/dl, females 11.9–14.3 g/dl

Haematrocrite and haemoglobin below, all other values above threshold

### Clinical chemistry

Results from clinical chemistry are summarized in Table 6. ASAT and gamma-GT were elevated in 13.3 and 17.8 % of the participants of group A, respectively. In the other groups, the proportion of increased values was 29.0 % (ASAT) and 38.7 % (gamma-GT) in group B and 0.0 % (ASAT) and 29.4 % (gamma-GT) in group C, respectively. For none of the variables, the percentage of values outside the normal range differed between the groups. Bilirubin and lipase were normal in all cases.

**Table 6:** Clinical chemistry in the three groups

	Group A (*n* = 45)		Group B (*n* = 31)		Group C (*n* = 18)	
Variable	Median (range)	Outside normal range^a^ (%)	Median (range)	Outside normal range^a^ (%)	Median (range)	Outside normal range^a^ (%)
ALP (U/l)	60 (27–133)	2/45 (4.4)	72 (34–111)	0/31 (0.0)	53 (30–84)	0/17 (0.0)
ALAT (U/l)	14.5 (5.6–51.9)	1/45 (2.2)	23 (11–54)	3/31 (9.7)	23 (12–54)	1/17 (5.9)
ASAT (U/l)	34 (16–123)	6/45 (13.3	36 (21–55)	9/31 (29.0)	30 (14–39)	0/17 (0.0)
Gamma GT (U/l)	28 (5–541)	8/45 (17.8)	38 (3–263)	12/31 (38.7)	23 (3–138)	5/17 (29.4)
Lipase (KU/l)	26.4 (15.1–46.9)	0/45 (0.0)	33 (18–80)	2/31 (6.5)	28 (15–81)	1/17 (5.9)
Bilirubine (mg/dl)	0.22 (0.04–1.16)	0/45 (0.0)	0.2 (0.1–1.1)	0/31 (0.0)	0.3 (0.2–0.7)	0/17 (0.0)

*ALAT* glutamate pyruvate transaminase (synonyms: alanine transaminase (ALT), serum glutamic pyruvic transaminase (SGPT), alanine aminotransferase), *ASAT* aspartate transaminase (synonyms: aspartate aminotransferase (AspAT/AST/AAT), serum glutamic oxaloacetic transaminase (SGOT), *Gamma GT* gamma-glutamyltransferase (synonyms: gamma-glutamyl transpeptidase (GGT, GGTP, gamma-GT)

^a^Measurements above threshold

## Discussion

*O. viverrini* infection is widely distributed in the four Southeast Asia countries: Thailand, Cambodia, Lao PDR and Vietnam, but the prevalence and the intensity of infection vary widely. Whereas in some areas in Northeast Thailand, prevalence and intensity of infection are extremely high [2]; in Cambodia and Lao PDR, intensity seems to be rather low [3, 5]. In Lahanam village, the prevalence in adults was 75 % by microscopic examination, but the intensity in the general population was low (Pongvongsa Tienkham, unpublished observation 2011). Presumably, prevalence and intensity of human infection essentially depend on eating customs [6, 10].

Since eating habits are deeply anchored in the culture of populations affected, they are difficult to change. Hence, efforts to control food-borne parasites are largely dependent on chemotherapy. The drug of choice for treatment of most food-borne trematodes is praziquantel. Because elimination of opisthorchiasis is currently not feasible, morbidity control through regular treatment with praziquantel is the only option.

There is general agreement that in a *O. viverrini*-infected population, only a minority carries a heavy disease burden, as indicated by an extremely high number of *O. viverrini* eggs per gram of stool (>10,000 epg) [1, 12]. Intensity of infection generally peaks in the middle age groups [12] and eggs per gram of faeces correlate to the number of flukes present in the bile duct, hence, are considered as an estimate for the intensity of infection [12].

In the participants of the highly endemic village Lahanam, located in the flood-prone lowland of Savannakhet Province, the excretion of *O. viverrini* eggs in stool ranged from 60 to 13,710 epg and from 1 to 7140 in duodenal fluid. Six patients (13.3 %) excreted more than 6000 epg, the threshold for heavy infection [13]. This indicates that the majority of the participants in group A were only lightly or moderately infected with *O. viverrini.* Fifty-three percent of the participants of group B and 50 % of group C excreted *O. viverrini* eggs in stool. However, the intensity of infection was lower by a factor 22 and 37, respectively, as compared to group A.

Potentially infectious fish dishes (raw fish with chilli sauce, raw fish mixed with vegetables and semi-fermented fish) were regularly eaten in group A and to a lesser extent in groups B and C. We think that in group B, consumption of fermented fish was limited by the fact that the village is located on a hilly plateau, and therefore, local people cannot catch fish by themselves such as it was the case in group A. Hence, people from group B had to buy fish food from dealers, and presumably, fermented fish was not regularly available. In group C, there was a tendency to preferably consume safer type of fish dishes, i.e. boiled, deep-fried or dried. The differences in the consumption of potentially infectious fish dishes explain the low and very low intensity of *O. viverrini* infection in groups B and C.

### Clinical pathology

Previous studies indicated that most chronically infected individuals present only few specific signs or symptoms, except an increased frequency of palpable liver [2, 8]. In fact, none of participants infected with *O. viverrini* showed signs of severe liver disease such as ascites, dilated abdominal veins, spider naevi or palmar erythema. The frequency of abdominal symptoms (nausea, vomiting, feeling of fullness, abdominal discomfort and indigestion) was generally low and similar in the three groups.

The average excretion of *O. viverrini* eggs in stool did not differ between participants with a low (≤10) and a high complaint score (>10 points). These observations corroborate previous findings in lightly to moderately infected patients [14, 15]. Similarly, in *C. sinensis* infection, 40 % of patients with light infection and 19 % with moderate infection had no symptoms [16]. Interestingly, in Italian patients heavily infected with *O. felineus*, only 10 % presented symptoms [17].

Population-based studies in Northeast Thailand clearly showed that the frequency and intensity of *O. viverrini-*associated symptoms are positively correlated with the intensity of infection [2]. Similar observations have been made in areas where *C. sinensis* is the predominant liver fluke [16]. Besides, Sripa did not observe pathological changes in the biliary epithelium and the periductal areas of liver tissue in light *O. viverrini* infections, whereas in severe infections, epithelial hyperplasia and periductal fibrosis were common [18]. It was, therefore, not surprising that ultrasonographic abnormalities were not detectable and that complaints were similar in the three groups. We also can exclude that due to an observer bias, bile duct abnormalities were overlooked. Ultrasonography was performed by an expert with more than 35 years of experience in- and outside the tropics. However, we have to acknowledge that—in order to allow the examination of the participants where they lived—a portable ultrasound device was used which probably had a lower resolution compared to the highly sophisticated equipment used in other studies [7, 8].

### Parasitological data

In group A, 15.6 % of the stool samples were positive for Taenia eggs, whereas in groups B and C, Taenia eggs were not observed at all. We explain this finding by the observation that in all Taenia positive stool samples, the concentration of Taenia eggs was at the limit of sensitivity of the Kato-Katz method. Hence, an unknown number of false-negative stool samples might have occurred in all groups.

### Biochemistry

Overall, biochemical parameters were similar in the three groups. ASAT and gamma-GT were elevated only in 13 and 18 % of the participants of group A, respectively, and the increase was rather low. Bilirubine was not elevated in a single case. The average excretion of *O. viverrini* eggs in stool did not differ between individuals with normal or elevated liver enzymes. This corresponds to previous findings in lightly to moderately infected patients [14, 16]. These authors also showed that an increase of liver enzymes and bilirubine is to be expected only in advanced liver fluke disease [16].

### Haematology

Adult flukes constantly ingest blood, and haemoglobin degradation products have been found in the worm gut [16]. Whether in heavy infection this results in anaemia is a matter of debate [16]. In participants in group A, anaemia was three times as frequent in males as compared to females. Excretion of *O. viverrini* eggs was also higher in males than in females, a finding consistent with data from population-based studies [1]. However, it remains speculative whether the lower haemoglobin values in male patients were due to the presence of a higher number of adult flukes in their bile ducts. It is more plausible that the anaemia was caused by the co-infection with hookworms (Table 4).

### Limitations

This study has a clear limitation. Since participants of groups B and C were enrolled in the study on a first-come-first-serve basis, a selection bias may have been introduced. Whether this resulted in the inclusion of a disproportionate high or low number of individuals with *O. viverrini* infection is impossible to say. We are also unable to provide evidence that the participants of groups B and C were truly representative for the community of Non Somboon and the staff of Savannakhet Hospital, respectively. The fact that we were unable to achieve the calculated sample size in groups B and C also hampers the interpretation of results.

## Conclusions

Taken together, the study shows that eating habits favouring the infection with *O. viverrini* are common in rural Laos. The participants of this study were only lightly to moderately infected with *O. viverrini* with only a few individuals carrying many worms. Clinically, this corresponded to a paucity of liver disease-associated complaints and signs. The generally low intensity of infection also explains why no alterations of bile ducts were detectable by ultrasonography.
